# Molecular characterization of *Cryptosporidium* spp. in Bactrian camels (*Camelus bactrianus*) from Yili Kazak Autonomous Prefecture of Xinjiang, China

**DOI:** 10.3389/fvets.2024.1411377

**Published:** 2024-06-10

**Authors:** Rongsheng Mi, Amanguli Silayi, Yongsheng Wang, Chenyang Xia, Wenqiang Tang, Haiyan Gong, Yan Huang, Yan Zhang, Genqiang Yan, Zhaoguo Chen

**Affiliations:** ^1^College of Animal Science and Technology, Shihezi University, Shihezi, China; ^2^Key Laboratory of Animal Parasitology of Ministry of Agriculture and Rural Affairs, Laboratory of Quality and Safety Risk Assessment for Animal Products on Biohazards (Shanghai) of Ministry of Agriculture and Rural Affairs, Shanghai Veterinary Research Institute, Chinese Academy of Agricultural Sciences, Shanghai, China; ^3^Yili Prefecture Center for Animal Disease Control and Diagnosis of Xinjiang, Yining, China; ^4^Tibet Academy of Agricultural and Animal Husbandry Sciences, Lhasa, China

**Keywords:** *Cryptosporidium* spp., molecular epidemiology, Bactrian camel, Yili Prefecture, Xinjiang

## Abstract

**Introduction:**

*Cryptosporidium* spp. is a significant zoonotic parasite. The prevalence and infection characteristics of *Cryptosporidium* spp. in Bactrian camels in Yili Kazak Autonomous Prefecture have yet to be fully understood. Thus, the molecular epidemiology of cryptosporidiosis in camels was investigated in this region.

**Methods:**

A total of 1,455 fecal samples were collected from 6 counties in three regions (Altay, Tacheng, and Yili) in Yili Prefecture. Nested PCR targeting the small subunit ribosomal RNA (*ssu* rRNA) gene was used to identify the species or genotypes of *Cryptosporidium* infection in camels. For *C. parvum* positive samples, the subtypes were identified using the 60-kDa glycoprotein (gp60) gene.

**Results and discussion:**

The overall infection rate was 8.7% (126/1,455), ranging from 5.6% to 11.7% in different regions, and 4.2% to 15.8% in different counties. A significant difference was observed amongst the counties (*p* < 0.001). Three species were detected, namely *C. andersoni* (65.1%, 82/126), *C. parvum* (34.1%, 43/126), and *C. occultus* (0.8%, 1/126). Three *C. parvum* subtypes, If-like-A15G2 (*n* = 29), IIdA15G1 (*n* = 4), and IIdA19G1(*n* = 1) were detected, with If-like-A15G2 being the most prevalent subtype. Camels aged 3-12 months exhibited the highest infection rate (11.4%, 44/387), with no significant difference among age groups (*p* > 0.05). *C. parvum* was predominant in camels under 3 months, while *C. andersoni* prevailed in camels over 3 months. There was an extremely significant difference observed among seasons (*p* < 0.001), summer had the highest infection rates (16.9%, 61/360). This study collected nearly 1,500 samples and, for the first time, investigated *Cryptosporidium* spp. infection in camels based on different age groups and seasons. All three *Cryptosporidium*species identified were zoonotic, posing a potential threat to human health and requiring close attention.

## 1 Introduction

*Cryptosporidium* spp. is one of the most common zoonotic parasites affecting humans and a broad range of animal species worldwide (1). It is a leading cause of persistent diarrhea in its hosts (1). The transmission of cryptosporidiosis primarily occurs through the fecal-oral route (2). In 2006, cryptosporidiosis as a neglected disease was listed by the World Health Organization (WHO) (3). At present, more than 45 species and 120 genotypes of *Cryptosporidium* have been identified using molecular detection methods. However, partial species can infect humans, and *C. hominis* and *C. parvum* are the most common species in humans (4). To date, there are no effective drugs or vaccines for the prevention and control of cryptosporidiosis (5). Therefore, it is very important to understand the route of transmission, and the species or genotypes of *Cryptosporidium* infection, which can provide data support for developing precise prevention and control strategies to reduce the damage to both humans and animals.

There are three *Camelus* species, namely *C. dromedarius* (the domesticated single-humped camel), *C. bactrianus* (the domesticated two-humped camel), and *C. ferus* (the wild two-humped camel) (6). Bactrian camels (*Camelus bactrianus*), as “ships of the desert,” are usually used as draft animals for transportation and riding. They are one of the important livestock resources in the desert and semi-desert areas of northwest and north China (7). Camels play an important role in the grassland animal husbandry of this region, holding a crucial position in the frontier animal husbandry and the ethnic characteristic industries of Mongolian and Kazak in the frontier pastoral areas (6). The camel breeding industry has transitioned from nomadic to intensive breeding, providing humans with milk, meat, camel hair, and other products, thereby increasing the economic income of herdsmen (8). While there have been limited global studies on *Cryptosporidium* spp. in camels. The first report of a Bactrian camel infected with *C. muris* was documented in United States in 1991 by Fayer et al. (9). Subsequent reports of *Cryptosporidium* infection in camels have been documented in various countries, including the United States (10, 11), Czech Republic (12–14), China (15–20), Iran (21–25), Egypt (26–28), Iraq (29), Saudi Arabia (30), Algeria (31–34) and Australia (35), with the infection rate ranging from 1.3 to 37.9% (Table 1).

**Table 1:** Prevalence and distribution of *Cryptosporidium* species/subtypes in camels in published reports.

Country	Isolate	Camel breed	No. sample	No. positive (%)^*^	*Cryptosporidium* species (no.)^#^	*Gp60* gene subtypes (no.)	References
United States	zoo	*C. bactrianus*	1	/	*C. muris* (1)	/	(9)
United States	zoo	*C. bactrianus*	1	/	*C. muris* (1)	/	(10)
United States	Veterinary hospital	*C. bactrianus/C. dromedarius*	77	1 (1.3)	/	/	(11)
Czech Republic	zoo	*C. bactrianus*	2	/	*C. andersoni* (2)	/	(12)
Czech Republic	zoo	*C. bactrianus*	2	/	*C. andersoni* (2)	/	(13)
Czech Republic	zoo	*C. bactrianus*	1	/	*C. muris* (1)	/	(14)
China	zoo	*C. bactrianus*	2	/	*C. andersoni* (2)	/	(15)
China	zoo	*C. bactrianus*	2	/	*C. andersoni* (2)	/	(16)
China	zoo	*C. dromedarius*	4	/	*C. andersoni* (2)	/	(17)
China	farm	*C. bactrianus*	40	6 (15.0)	*C. andersoni* (4), *C. parvum* (2)	/	(18)
China	farm	*C. bactrianus*	476	36 (7.6)	*C. andersoni* (24), *C. parvum* (6), *C. ubiquitum* (2), *C. occultus* (2), *C. bovis* (1), *C. hominis* (1)	If-like-A15G2 (5); IIdA15G1 (1); IkA19G1 (1); XIIa (2)	(19)
China	zoo	*C. bactrianus*	2	/	*C. muris* (1)	/	(20)
Iran	Slaughter-house	*C. dromedarius*	103	39 (37.9)	/	/	(21)
Iran	farm	*C. dromedarius*	65	11 (16.9)	/	/	(22)
Iran	Slaughter-house	*C. dromedarius*	300/100^§^	61 (20.3)/24 (24.0)	/	/	(23)
Iran	farm	*C. dromedarius*	170	17 (10.0)	/	/	(24)
Iran	farm	*C. dromedarius*	85	2 (2.4)	*/*	/	(25)
Egypt	Slaughter-house	*C. dromedarius*	145	28 (19.3)	*C. muris* (1)	/	(26)
Egypt	Slaughter-house	*C. dromedarius*	101	6 (5.9)	*C. parvum* (2), Rat genotype IV (1), Camel genotype (3)	IIdA19G1 (1), IIaA15G1R1 (1)	(27)
Egypt	farm	*C. dromedarius*	102	3 (2.9)	*C. parvum* (2), *C. bovis* (1)	/	(28)
Iraq	farm	*C. dromedarius*	100	61 (61.0)	*/*	/	(29)
Saudi Arabia	farm	*C. dromedarius*	33	5 (15.1)	*/*	/	(30)
Algeria	farm	*C. dromedarius*	39	2 (5.1)	*C. parvum* (2)	If-like A22G2 (2)	(31)
Algeria	farm	*C. dromedarius*	149	3 (2.0)	*/*	/	(32)
Algeria	farm	*C. dromedarius*	717	13 (1.8)	/	/	(33)
Algeria	farm	*C. dromedarius*	63	5 (7.9)	*C. parvum* (4), *C. bovis* (1)	IIaA15G2R1 (1), IIaA17G2R1 (1), IIaA15G2R1 + IIaA18G2R1 (1), IIdA19G1 (1)	(34)
Australian	wild	*C. dromedarius*	1	/	*C. parvum* (1)	IIaA17G2R1 (1)	(35)

^*^ Partial positive samples from zoo or wild camels, not involving infection rates. ^#^ Partial samples examined by microscopic method, species were not identified. ^§^ 300 samples from feces and 100 samples from mucosa.

However, research on camels infected with *Cryptosporidium* spp. in China is scarce. The first report of two camels infected with *C. andersoni* in China was in 2008 from a zoo in Henan Province by Wang et al. (15). Since then, subsequent studies on *Cryptosporidium* infection in camels have been reported in provinces such as Sichuan (16), Anhui (17), Qinghai (18), Xinjiang (19), and Henan (20) with the majority found in zoos (15–17, 20) and limited sample sizes. Only two studies have reported prevalence rates of *Cryptosporidium* in camels, with 15.0% (6/40) in Qinghai (18) and 7.6% (36/476) in Xinjiang (19), respectively. The infected species included *C. andersoni*, *C. bovis*, *C. parvum*, *C. occultus*, *C. ubiquitum*, *C. muris*, and *C. hominis* (18, 19). Subtypes identified were If-like-A15G2 and IIdA15G1 of *C. parvum*, XIIa of *C. ubiquitum*, and IkA19G1 of *C. hominis* (19).

The Yili Kazakh Autonomous Prefecture (Yili Prefecture) is situated in the northwest of Xinjiang, spanning from 80°09′E to 91°01′E longitude and 40°14′N to 49°10′N latitude. There are three regions in Yili Prefecture, including Altay region, Tacheng region and Yili region. According to the National Bureau of Statistics of China,^1^ the total number of camels was 461,700 at the end of 2021, and Xinjiang has the largest population of camels, accounting for 48.7% (about 225,600) in China. In terms of Xinjiang, Yili Prefecture has the largest amount of camels.^2^ Despite being a significant parasite causing diarrhea, the current status of *Cryptosporidium* spp. infection in camels remains unclear. Therefore, the objective of this study is to conduct the molecular characterization of *Cryptosporidium* in camels in Yili Prefecture, aiming to provide data support for the prevention and control of *Cryptosporidium* in camels in this region.

## 2 Materials and methods

### 2.1 Sample collection

Fecal samples were collected from three regions in Yili Prefecture, and two counties in each region, which included Tuoli County (83°40′E, 45°55′N) and Yumin County (83°00′E, 46°05′N) in Tacheng region, Fuyun County (89°27′E, 46°59′N) and Fuhai County (87°23′E, 47°07′N) in Altay region, and Huocheng County (80°50′E, 44°03′N) and Yining County (81°34′E, 43°58′N) in Yili region. One farm was selected from each county. Samples were collected from each farm across four seasons (spring, summer, autumn, and winter), with each sampling season involving sampling across four age groups (<3 months old, 3–12 months old, 1–2 years old, and > 2 years old), and at least 15 samples were collected from each age group. Most camels exhibited no noticeable symptoms, except for a few that showed mild diarrhea. Camels under 3 months old primarily relied on milk for nutrition, gradually started to eat grass over 4 months old, and weaned around 1 year old. Grazing was the primary activity from May to October, and house-feeding was common from November to April. Camels were grazing on the Gobi Desert plains every morning and went back in the evening. Camels under 3 months old were primarily fed in the breeding houses. Water was provided once a day in spring and autumn, 1 to 2 times a day in summer, and every other day in winter. Fresh fecal samples were collected either from the ground or via rectal sampling and were stored in labeled 10 mL centrifuge tubes along with information, such as sampling time, location, and age before being refrigerated and transported back to the laboratory. To prevent cross-contamination, when fresh fecal samples were collected from the ground, the samples were taken on the surface and avoided contact with the ground.

### 2.2 Sample processing and DNA extraction

Fecal samples (about 300 mg) were taken to a 5 mL centrifuge tube, added 2 mL phosphate-buffered saline (PBS) in the tube. Following thorough mixing of the contents by repeatedly pipetting, and two washes, 1 mL of the sample was transferred to a 2 mL sterile centrifuge tube for DNA extraction using FastDNA SPIN Kit for Soil (MP Biomedicals, Santa Ana, CA). Extracted DNA was stored at −20°C until further analysis.

### 2.3 PCR amplification

Nested PCR targeting the small subunit ribosomal RNA (*ssu* rRNA) gene was utilized to identify *Cryptosporidium* species. The primers used for the primary PCR were 5′-TTC TAG AGC TAA TAC ATG CG-3′ (*ssu*-F1) and 5′-CCC ATT TCC TTC GAA ACA GGA-3′ (*ssu*-R1), while the second PCR primers were 5′-GGA AGG GTT GTA TTT ATT AGA TAA AG-3′ (*ssu*-F2) and 5′-CTC ATA AGG TGC TGA AGG AGT A-3′ (*ssu*-R2) (10, 36). The primary PCR reaction contains were as follows: 25.0 μL of 2 × PCR buffer, 8.0 μL of dNTP (2 mmol/L), 1.0 μL of 1 U KOD FX (Toyobo, Japan) *Taq* polymerase, 0.5 μL (10 μmol/L) of each forward (*ssu*-F1) and reverse (*ssu*-R1) primer, 2.0 μL of BSA (20 mg/mL), 1.0 μL of DNA template, and added H_2_O up to 50.0 μL. For the second PCR system, 1 μL of primary PCR product was used as a DNA template, and the primers were changed to *ssu*-F2 and *ssu*-R2, other conditions were the same as the primary PCR. Each PCR reaction included positive and negative controls. The PCR amplification conditions comprised pre-denaturation at 94°C for 5 min; denaturation at 94°C for 45 s, annealing at 56°C for 45 s, extension at 72°C for 1 min, 30 cycles; extension at 72°C for 10 min. The second PCR amplification conditions were the same as the primary PCR, except for the annealing temperature at 60°C. After the PCR amplification, 5 μL of the second PCR products (about 830 bp) were identified by agar gel electrophoresis.

### 2.4 Sequences analysis

All positively confirmed samples by electrophoresis were sequenced by Sangon Biotech (Shanghai) Co., Ltd. The obtained sequences were analyzed through Blast alignment in NCBI^3^ for *Cryptosporidium* species identification in camels. Relevant *ssu* rRNA sequences of *Cryptosporidium* spp. were downloaded from GenBank, and phylogenetic tree analysis was performed using Molecular Evolutionary Genetics Analysis (MEGA) 11.0 software^4^ to further confirm the species or genotypes of *Cryptosporidium* in camels.

### 2.5 Subtype identification of *C. parvum* positive samples

*Cryptosporidium parvum-*positive samples were subjected to subtype identification using the 60-kDa glycoprotein (*gp60*) gene as the target. The primary PCR primers were 5′-ATA GTC TCC GCT GTA TTC-3′ (*gp60*-F1) and 5′-GGA AGG AAC GAT GTA TCT-3′ (*gp60*-R1). The second PCR primers were 5′-TCC GCT GTA TTC TCA GCC-3′ (*gp60*-F2) and 5′-GCA GAG GAA CCA GCA TC-3′ (*gp60*-R2) (37, 38). The sample preparation and amplification conditions for PCR were consistent with those for the *ssu* rRNA gene. Positive samples underwent sequencing (about 850 bp), and subtype analysis was performed following previously reported methods (39).

### 2.6 Statistical analysis

The differences among different counties, ages, and seasons were performed chi-square tests (*χ^2^*) for *p* value by IBM SPSS 21.0 software (IBM Corp., Armonk, NY, USA). No differences among different factors when *p* > 0.05, significant differences when *p* < 0.05, and extremely significant differences were judged when *p* < 0.001.

## 3 Results

### 3.1 The overall infection of *Cryptosporidium* spp. in camels

**Figure 1:**
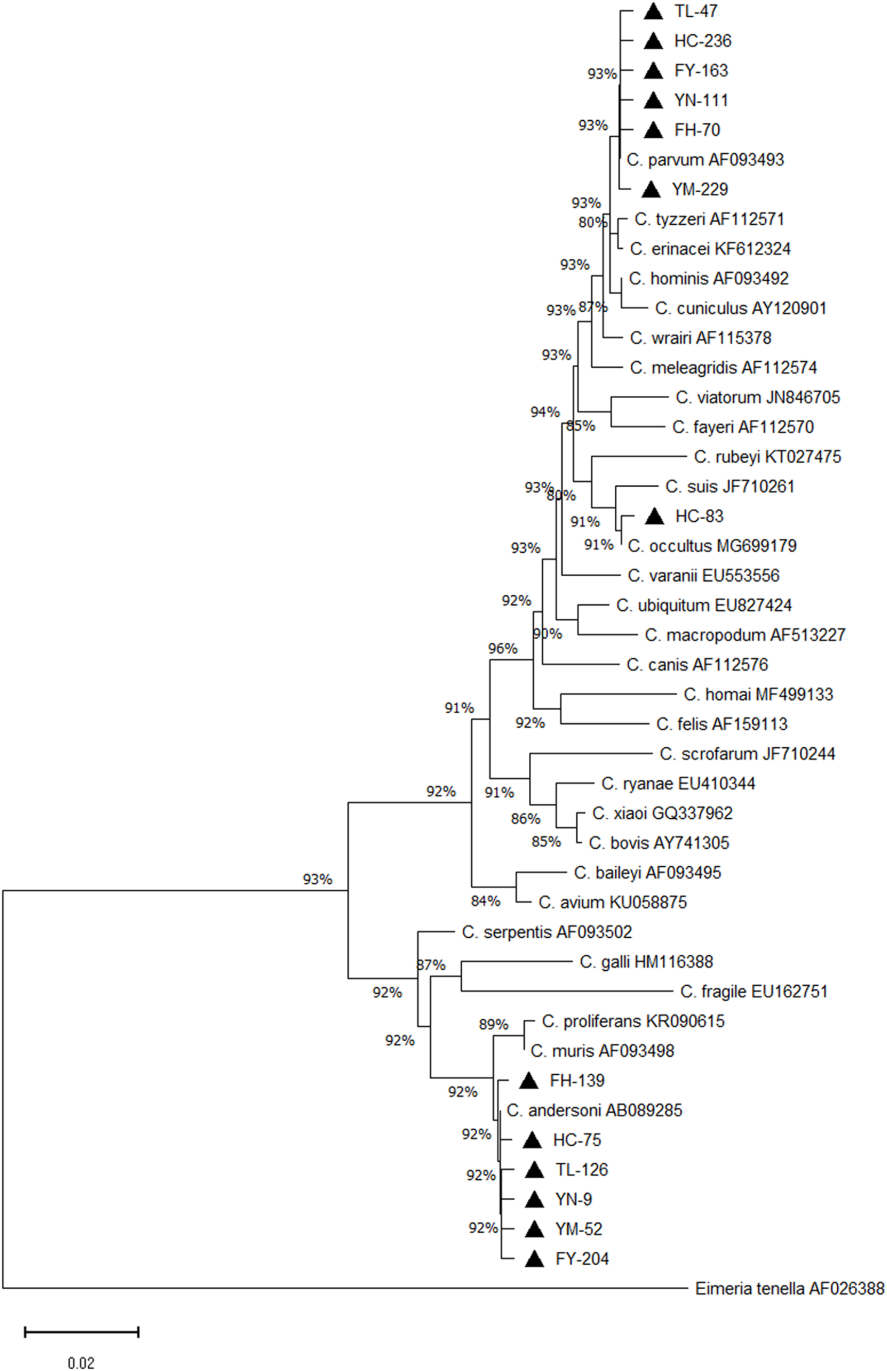
Phylogenetic tree of *Cryptosporidium* spp. Constructed based on *ssu* rRNA nucleotide sequence in camels. ▲: Partial sequences obtained in the present study.

Of 1,455 samples collected, 126 tested positive, resulting in an 8.7% positivity rate (95%CI, 7.3–10.2). Sequencing of positive samples revealed three *Cryptosporidium* species in camels: *C. andersoni*, *C. parvum*, and *C. occultus*. *C. andersoni* was the predominant species, accounting for 65.1% (82/126) of the positive samples, followed by *C. parvum* at 34.1% (43/126). Additionally, one case of *C. occultus* infection was found, accounting for 0.8% (1/126) (Table 2). A phylogenetic tree was constructed using MEGA software for *Cryptosporidium* species identification (Figure 1).

**Table 2:** The prevalence of *Cryptosporidium* spp. in different regions.

Region	No. Sample	No. Positive (%)	95% CI	*p* value	Species		
					*C. parvum*	*C. andersoni*	*C. occultus*
Tacheng	480	56 (11.7)	8.9–14.9	0.001*	27	29	0
Altay	494	43 (8.7)	6.4–11.6	0.062	13	30	0
Yili	481	27 (5.6)	3.7–8.1	Ref.	3	23	1
Total	1,455	126 (8.7)	7.3–10.2		43	82	1

* Represents significant (*p* < 0.05).

### 3.2 The infection of *Cryptosporidium* spp. in different regions

In this study, samples were collected from three regions in Yili Prefecture. The infection rate of *Cryptosporidium* spp. was 11.7% (27/481; 95%CI, 8.9–14.9) in Tacheng region, 8.7% (43/494; 95%CI, 6.4–11.6) in Altay region, and 5.6% (27/481; 95%CI, 3.7–8.1) in Yili region (Table 2). The overall infection rates showed significant differences among different regions (*x^2^* = 11.132, *p* < 0.05). While, there were no significant differences between the Altay region or Yili or Tacheng region, an extremely significant difference was noted between Yili and Tacheng region (*x^2^* = 11.157, *p* < 0.001).

As the most common species, *C. andersoni* was detected in the Yili region (85.2%, 23/27) and the Altay region (69.8%, 30/43). While a similar number of *C. parvum* (*n* = 27) and *C. andersoni* (*n* = 29) were identified in the Tacheng region. The infection rate of *C. parvum* in the Tacheng region (62.8%, 27/43) was significantly higher than that in the Altay region (30.2%, 13/43) and Yili region (7.0%, 3/43) (Table 2).

### 3.3 The infection of *Cryptosporidium* spp. in different counties

**Figure 2:**
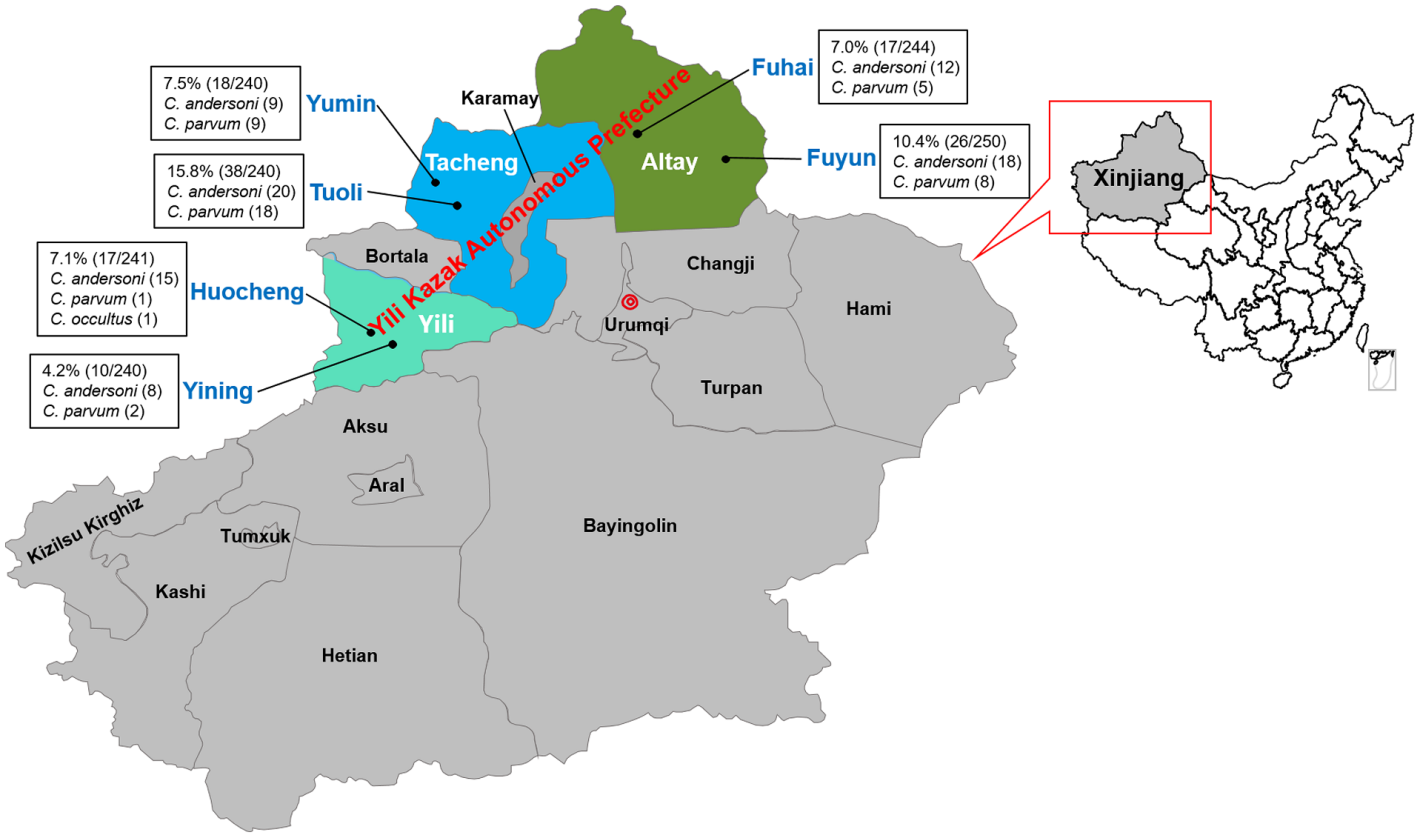
Map of sample collection sites for camels in Yili Prefecture. ● Samples collected sites. The data for the underlying map were downloaded from the Database of Global Administrative Areas (GADM) website (https://gadm.org/maps/CHN/xinjianguygur_2.html) and revised with PowerPoint (Microsoft Office 2021).

The study’s samples spanned six counties across three regions. The results showed that the highest infection rate was found in Toli County, reaching 15.8% (38/240; 95% CI, 11.5–21.1), and the lowest in Yining County, at 4.2% (10/240; 95% CI, 2.0–7.5). The infection rates in Huocheng County, Fuhai County, and Yumin County were similar, at 7.1% (17/241; 95% CI, 4.2–11.1), 7.0% (17/244; 95% CI, 4.1–10.9), and 7.5% (18/240; 95% CI, 4.5–11.6) respectively, while the infection rate in Fuyun County was slightly lower than that in Toli County, at 10.4% (26/250; 95% CI, 6.9–14.9) (Table 3 and Figure 2). Statistical analysis showed an extremely significant difference in the overall infection rates between different counties (*x^2^* = 24.774, *p* < 0.001). However, the differences between counties were not consistent. The infection rate between Toli County and Fuyun County was not significant (*x^2^* = 3.183, *p* > 0.05), but there was a significant difference between Toli County and the other four counties (*p* < 0.05).

**Table 3:** The prevalence of *Cryptosporidium* spp. in different counties.

Region	County	No. Sample	No. Positive (%)	95% CI	*p* value	Species		
						*C. parvum*	*C. andersoni*	*C. occultus*
Tacheng	Tuoli	240	38 (15.8)	11.5–21.1	<0.001*	18	20	0
	Yumin	240	18 (7.5)	4.5–11.6	0.004*	9	9	0
Altay	Fuyun	250	26 (10.4)	6.9–14.9	0.074	8	18	0
	Fuhai	244	17 (7.0)	4.1–10.9	0.002*	5	12	0
Yili	Huocheng	241	17 (7.1)	4.2–11.1	0.002*	1	15	1
	Yining	240	10 (4.2)	2.0–7.5	Ref	2	8	0
Total		1,455	126 (8.7)	7.3–10.2		43	82	1

* Represents significant (*p* < 0.05).

*Cryptosporidium andersoni* exhibited the highest infection rate in Yining (80.0%, 8/10), Huocheng (93.8%, 15/16), Fuhai (70.6%, 12/17) and Fuyun (69.2%, 18/26) counties. Similar infection numbers of *C. andersoni* and *C. parvum* were found in the Yumin and Tuoli counties of the Tacheng region. As a unique species, *C. occultus* was identified in Huocheng County of Yili region (Table 3 and Figure 2).

### 3.4 The infection of *Cryptosporidium* spp. in different ages

The survey included samples from four age groups. The highest infection rate was observed in camels aged 3–12 months, at 11.4% (44/387; 95%CI, 8.4–15.0), followed by camels over 2 years old, at 8.0% (35/437; 95%CI, 5.6–11.0). Similar infection was detected in camels under 3 months and those aged 1–2 years, at 9.3% (25/269, 95%CI, 6.1–13.4) and 6.1% (22/362, 95%CI, 3.9–9.1), respectively (Table 4). A significant difference was observed between camels aged 3–12 months and 1–2 years (*x^2^* = 6.519, *p* < 0.05), while the infection rates in other age groups showed no significant difference (*p* > 0.05).

**Table 4:** The prevalence of *Cryptosporidium* spp. in different age groups.

Age	No. Sample	No. Positive (%)	95% CI	*p* value	Species		
					*C. parvum*	*C. andersoni*	*C. occultus*
<3 months	269	25 (9.3)	6.1–13.4	0.322	17	7	1
3–12 months	387	44 (11.4)	8.4–15.0	0.065	12	32	0
1–2 years	362	22 (6.1)	3.9–9.1	0.180	8	14	0
>2 years	437	35 (8.0)	5.6–11.0	Ref.	6	29	0
Total	1,455	126 (8.7)	7.3–10.2		43	82	1

*Cryptosporidium parvum* and *C. andersoni* were found across all age groups. *C. parvum*, the highest infection species, was found in camels under 3 months (68.0%, 17/25). In contrast, *C. andersoni* was predominant in camels aged 3–12 months (72.7%, 32/44), 1–2 years (63.6%, 14/22), and over 2 years (82.9%, 29/35) age groups. *Cryptosporidium occultus* was detected in camels under 3 months (Table 4).

### 3.5 The infection of *Cryptosporidium* spp. in different seasons

The highest infection rate was observed in summer, at 16.9% (61/360; 95%CI, 13.2–21.2), followed by spring at 8.3% (30/320; 95%CI, 5.7–11.7), winter at 6.3% (23/364; 95%CI, 4.1–9.3), and autumn exhibiting the lowest infection rate at 3.2% (12/371; 95%CI, 1.7–5.6) (Table 5). Extremely significant differences were observed between different seasons (*x^2^* = 47.614, *p* < 0.001), with summer showing an extremely significant difference compared to other seasons (*p* < 0.001). However, the difference between spring and winter was not significant (*p* > 0.05).

**Table 5:** The prevalence of *Cryptosporidium* spp. in different seasons.

Season	No. Sample	No. Positive (%)	95% CI	*p* value	Species		
					*C. parvum*	*C. andersoni*	*C. occultus*
Spring	360	30 (8.3)	5.7–11.7	0.185	10	20	0
Summer	360	61 (16.9)	13.2–21.2	<0.001*	24	37	0
Autumn	371	12 (3.2)	1.7–5.6	0.036*	6	5	1
Winter	364	23 (6.3)	4.1–9.3	Ref.	3	20	0
Total	1,455	126 (8.7)	7.3–10.2		43	82	1

* Represents significant (*p* < 0.05).

Both *C. andersoni* and *C. parvum* were detected across all four seasons. *Cryptosporidium andersoni* as the predominant species were found in winter (87.0%, 20/23), spring (66.7%, 20/30), and summer (60.7%, 37/61). However, similar numbers of *C. parvum* (*n* = 6) and *C. andersoni* (*n* = 5) were found in autumn. The unique species of *C. occultus* was also detected in autumn (Table 5).

### 3.6 The subtypes of *C. parvum* in camels

**Figure 3:**
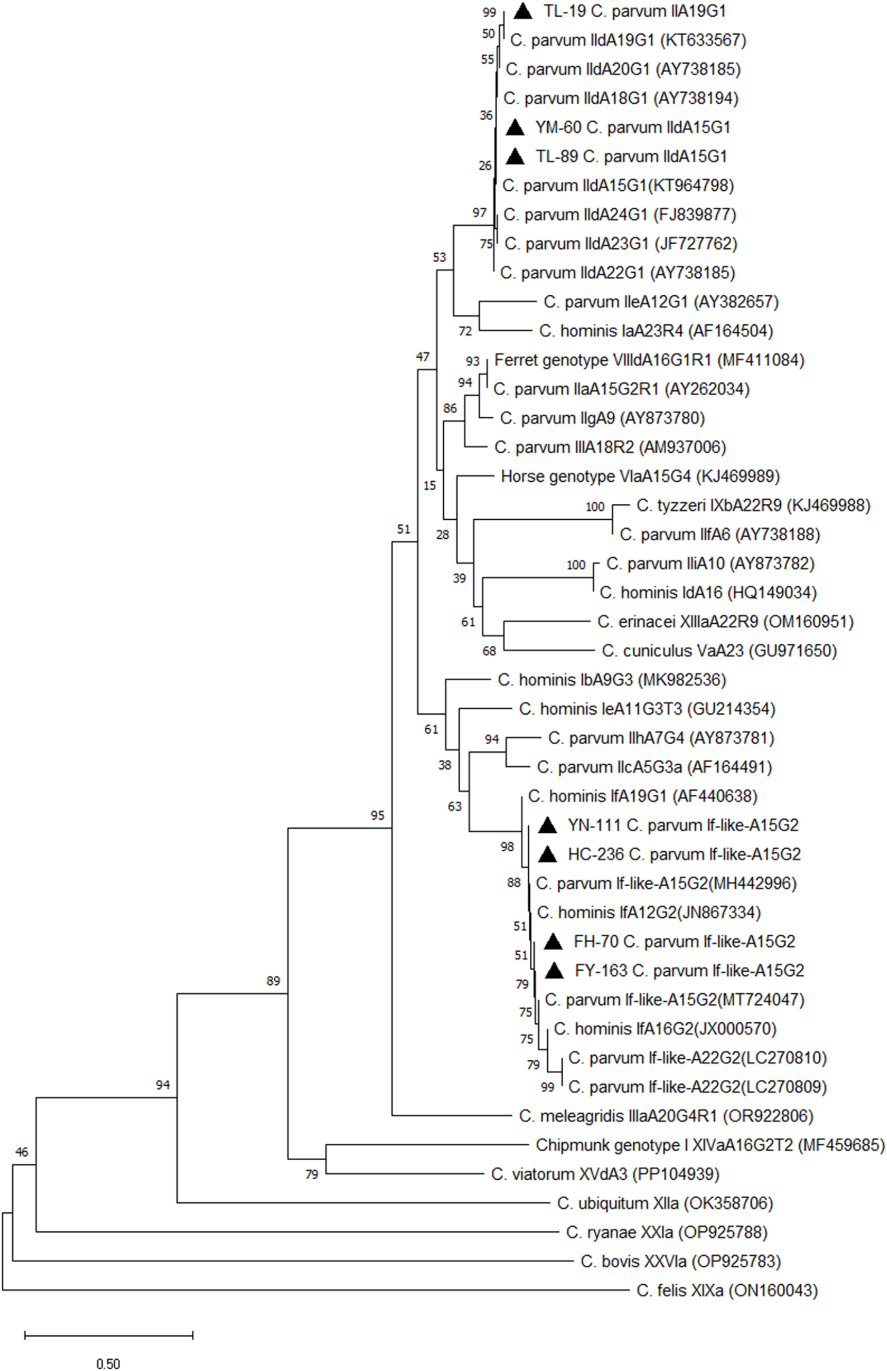
Phylogenetic tree of *Cryptosporidium* spp. Constructed based on *gp60* nucleotide sequence in camels. ▲: Partial sequences obtained in the present study.

In this study, all positive samples of *C. parvum* (*n* = 43) were subtyped using the *gp60* gene. Thirty-four *C. parvum*-positive samples were subtyped successfully. Three subtypes were detected, including If-likeA15G2 (85.3%, 29/34), IIdA15G1 (11.8%, 4/34) and IIdA19G1 (2.9%, 1/34). Subtype If-likeA15G2 was the most common subtype detected in all counties. The IIdA15G1 was identified in Yumin County (*n* = 2), Tuoli County (*n* = 1), and Fuhai County (*n* = 1). The unique subtype IIdA19G1 was detected in Tuoli County (Table 6). A phylogenetic tree was constructed based on the *gp60* gene of *Cryptosporidium* spp. for subtype identification (Figure 3).

**Table 6:** *Cryptosporidium parvum* subtype identification.

Region	County	*C. parvum* positive (no.)	Subtype successful (no.)	*C. parvum* subtypes (no.)
Tacheng	Tuoli	18	14	If-like-A15G2 (12), IIdA15G1 (1), IIdA19G1 (1)
	Yumin	9	7	If-like-A15G2 (5), IIdA15G1 (2)
Altay	Fuyun	8	6	If-like-A15G2 (6)
	Fuhai	5	5	If-like-A15G2 (4), IIdA15G1 (1)
Yili	Huocheng	1	1	If-like-A15G2 (1)
	Yining	2	1	If-like-A15G2 (1)
Total		43	34	If-like-A15G2 (29), IIdA15G1 (4), IIdA19G1 (1)

## 4 Discussion

*Cryptosporidium* spp. can infect a wide range of hosts, including humans, livestock, and wildlife, and spread through the fecal-oral route, which is contaminated with oocysts in water, food, or environmental surfaces (40). Oocysts are resistant to most disinfectants, and there are no effective drugs or vaccines to treat or prevent cryptosporidiosis in humans and animals (41). Therefore, cutting off the spread of *Cryptosporidium* between humans and animals is an important way to control *Cryptosporidium* infection (5). Investigating the infection situations and the dominant species or genotypes of *Cryptosporidium* in humans and animals is important to effectively control *Cryptosporidium* transmission. In addition, the epidemiological data, combined with the One Health approach, will help us develop effective plans for the prevention and control of cryptosporidiosis (42). While, as the most important livestock in the desert and semi-desert areas of the Middle East and China, the infection of *Cryptosporidium* spp. in camels is not clear.

Currently, only a few studies have documented *Cryptosporidium* infection in camels worldwide, with prevalence ranging from 1.3 to 37.9%, demonstrating significant variations in infection rates (Table 1). In this study, the prevalence of *Cryptosporidium* spp. infection in camels was examined in Yili Prefecture. The findings showed a prevalence of 8.7% (126/1,455) *Cryptosporidium* spp. in camels in Yili Prefecture, which exceeded previous reports from Algeria, where infection rates ranged from 1.8% (13/717) to 7.9% (5/63) (31–34), but were lower than previous reports in Iraq (29) and Saudi Arabia (30), with infection rate of 61.0% (61/100) and 15.1% (5/33), respectively. On the contrary, the infection rates we tested were higher than previous reports from Iran (2.4%) (25) and Egypt (2.9 and 5.9%) (27, 28), yet lower than other studies in the same countries, where the prevalence ranged from 10.0% (17/170) to 37.9% (39/103) in Iran (21–24), and 19.3% (28/145) in Egypt based on microscopic examination. The differences in these results may be related to geographical location, detection method, or camel breed.

In China, limited studies have been reported on *Cryptosporidium* infection in camels, most of which were related to a few samples (*n* < 10) in zoos (15–17, 20). Only two studies have focused on *Cryptosporidium* prevalence in camel farms, with a prevalence of 15.0% (6/40) in Qinghai Province (18), and 7.6% (36/476) in Xinjiang Uygur Autonomous Region (19). The infection rate (8.7%) in this study was lower than previous reports in Qinghai (18), but similar to Xinjiang (19), indicating that geographical location may be related to *Cryptosporidium* infection in camels. So far, only one study with limited samples of camels from Yili has been conducted, encompassing Tarbagatay District (*n* = 16) in the Tacheng region, Qapqal Xibe Autonomous County (*n* = 12) in the Yili region, Qinghe County (*n* = 57) and Fuhai County (*n* = 26) in the Altay region (19). Their results showed that the total infection rate was 7.2% (8/111) in Yili Prefecture (19), consistent with our findings. Subsequently, within the three regions, the infection rates were 18.8% (3/16) in the Tacheng region and 33.3% (4/12) in the Yili region (19), sharply higher than our results for the same regions. On the contrary, the prevalence in the Altay region was 1.2% (1/83) (19), obviously lower than our study. Notably, the prevalence in Fuhai County was 7.0% (17/244) in this study, surpassing the previous study (3.8%, 1/26) within the same county (19). Discrepancies between our study and previous research may be attributed to differences in sample size, collection seasons, and animal ages, as our study encompassed a broader range of fecal samples across various age groups and seasons.

Nowadays, six *Cryptosporidium* species and two genotypes have been reported in camels, comprising *C. muris*, *C. parvum*, *C. bovis*, *C. andersoni*, *C. ubiquitum*, *C. occultus*, *C. hominis*, *Cryptosporidium* rat genotype IV, and *Cryptosporidium* camel genotype (Table 1). Our study identified *C. andersoni*, *C. parvum*, and *C. occultus* in camels in Yili Prefecture, consistent with previous studies on the *Cryptosporidium* species. Additionally, *C. andersoni* exhibited the highest prevalence of species in Yili Prefecture (65.1%, 82/126), similar to previous studies in Qinghai and Xinjiang (18, 19). Therefore, it seems that *C. andersoni* is the predominant parasite species infecting camels in China, with several zoo studies also corroborating *C. andersoni* as the most prevalent species in China (15–19). On the contrary, *C. parvum* was the dominant species in North Africa, such as Egypt and Algeria (27, 28, 31, 34). Due to the limited studies on *Cryptosporidium* species in camels, this conclusion still requires validation through a large number of camel samples in the world.

A previous study in Yili Prefecture revealed *C. andersoni* as the unique species in Tarbagatay (*n* = 3) of the Tacheng region and in Qapqal Xibe (*n* = 4) of the Yili region, respectively, while *C. parvum* was the unique species in Fuhai (*n* = 1) of the Altay region (19). Contrasting with these findings, our study detected both *C. andersoni* and *C. parvum* in three regions, possibly due to the larger number of fecal specimens tested. Additionally, we identified a sample as *C. occultus* in Yili Prefecture for the first time, with this species also reported in camels in Qira County of Xinjiang by Cao et al. (19).

This study represents the first investigation of *Cryptosporidium* spp. infections in camels at different age groups in China. Consistent with previous studies indicating that young camels are more susceptible to *Cryptosporidium* infection (24, 28), the highest infection rate was found under 1 year of age. The prevalence in camels under 1 year was 10.5% (69/656), lower than the previous study of 20.0% (9/44) in Egypt (24). Another study found the highest prevalence was 15.4% under 2 years old (2/13) (28), while the prevalence was 8.9% (91/1,018) in this study. In addition, the prevalence in camels over 2 years old was 8.0% (35/437) in our study, higher than previous studies in Egypt, with the prevalence at 6.3% (8/126) and 1.1% (1/89) (24, 28). In contrast, high infection rates were reported in different age groups in Iran (23), Egypt (26), and Iraq (29). The prevalence was 19.2% (24/125) under 5 years old, 20.7% (29/140) in 5–10 years old, and 22.9% (8/35) over 10 years old in Iran (23); 66.7% (28/42) under 3 years old, 75.0% (18/24) in 3–6 years old, and 29.4% (10/34) over 6 years old in Iraq (29); and 19.3% (28/145) in 5–8 years old in Egypt (26). In addition, one study found that the prevalence was 7.7% (2/26) under 5 years old, 10.0% (2/20) in 5–10 years old, and no *Cryptosporidium* infection over 10 years old (0/10) in Algeria (30). The variations in infection rates among these studies may be related to geographical differences, camel breeds (dromedary camels in North Africa and the Middle East, and Bactrian camels in our study), or the number of samples collected. Since camels are weaned at 1 year old and juveniles at 1–2 years old, they then reach sexual maturity at 3–4 years old (43), while the samples collected over 2 years old were very small in this study and could not reflect the real infection characteristic of *Cryptosporidium* species in this age group, and a detailed age grouping over 2 years old is needed in future research. In addition, further study is needed to investigate whether there are differences in *Cryptosporidium* infection in camels between different genders.

Our, study represents the first investigation of the prevalence of *Cryptosporidium* across four seasons. Currently, there is limited research on the seasonal effects of camels, only one study showed similar infection rates in summer (18.0%, 27/150) and winter (22.7%, 34/150) in Iran (23). In contrast, an extremely significant difference (*p* < 0.001) was detected in camels among different seasons in this study, with the infection rate in summer significantly higher than in other seasons (*p* < 0.05). This difference may be related to the climate of Yili Prefecture, which is located far from the ocean and surrounded by mountains, exhibiting typical arid climatic characteristics and belonging to a semi-arid continental climate. The overall climatic features of the entire prefecture include short summers and long winters; rapid but unstable temperature rises in spring; and swift temperature drops in autumn. There is more rainfall and the highest temperatures in summer, with average temperatures above 20°C.^5^ These climatic features have significant impacts on the ecological environment, agricultural production, and local way of life in the Yili Prefecture, providing a unique backdrop for its culture and lifestyle. Few previous studies have shown that climate variability can affect the transmission of *Cryptosporidium* (44). However, it cannot be conclusively determined at present whether the infection of *Cryptosporidium* in camels is related to the season and further research is necessary.

According to a previous subtyping method (39), three *C. parvum* subtypes were identified in our study, including If-like-A15G2, IIdA19G1, and IIdA15G1, which was consistent with the previous study by Cao et al. (19), who found If-like-A15G2 and IIdA15G1 in Xinjiang. Additionally, a *C. hominis* subtype IkA19G1 and two *C. ubiquitum* subtypes XIIa were also detected in Xinjiang in their study (19). However, a different subtype of IIdA19G1 was discovered in this study, similar to previous studies in Egypt (27) and Algeria (34), where one IIdA19G1 subtype was found in each country. The most prevalent subtype of If-like-A15G2 was first reported in Fuhai County and Shihezi City in Xinjiang by Cao et al. (19), and another study found an If-like-A22G2 in dromedary camels in Algeria (31). The phylogenetic analysis found that both of these two If-like subtypes and *C. hominis* If subtypes were gathered in the same clade (19). These results indicated that *C. parvum* If-like subtypes showed a higher homology with *C. hominis* If subtypes. In addition, *C. parvum* IIa subtypes of IiaA15G1R1, IiaA17G2R1, and IiaA18G2R1 have been reported in Egypt (27), Algeria (34) and Australia (35) in dromedary camels, but we did not find them in our study.

All three species detected in camels in this study are zoonotic. *C. parvum* is the most common species in human cryptosporidiosis, with a broad range of hosts, including primates, ruminants, equine animals, and rodents (45). As the gastric *Cryptosporidium* species, *C. andersoni* is commonly reported in ruminants, particularly in adult cattle. Although *C. andersoni* has been reported in humans (46–48), the zoonotic transmission of *C. andersoni* between animals and humans still needs further research (46). *C. occultus*, previously named *Cryptosporidium suis*-like, was considered a valid species in 2018 by Kváč et al. (49). Recent studies found that *C. occultus* is a zoonotic species that is detected in many hosts, including humans, ruminants, and rodents (49–51). Previous studies showed that one of the significant factors for human cryptosporidiosis is the shedding of *Cryptosporidium* oocysts into the environment from livestock, and the most important transmission routes are food and water sources (1). Both *C. andersoni* and *C. parvum*, which were detected in this study, have been reported in water sources (52, 53), which suggested that water may be the transmission route for *Cryptosporidium* infection in camels. In addition, *C. occultus*, identified in this study, is a major rodent species (49). Given the many wild rodents in Yili Prefecture, camels infected with *C. occultus* may be from rodents that camels contacted with *Cryptosporidium* oocysts excreted by rodents during grazing. However, this study did not investigate the *Cryptosporidium* infection in farmers, water sources, or wild rodents, and the transmission pathways of *Cryptosporidium* in camels in this region remain unclear. Further research is needed to investigate the infection of *Cryptosporidium* in farmers, wild animals, and the surrounding environment in this area, and combine it with the One Health approach for cryptosporidiosis prevention and control in camels.

Cryptosporidiosis is a global distribution disease with self-limiting diarrhea, abdominal pain, decreased appetite, vomiting, and weight loss (1). However, the clinical symptoms in camels are not clear currently. In this study, except for a few camels under 3 months old who experienced mild diarrhea, the other camels did not show obvious clinical symptoms. This result suggests that most of the *Cryptosporidium* in camels are asymptomatic infections. Unfortunately, due to the limited number of diarrhea samples, we did not label these samples separately, and the relationship between diarrhea samples and *Cryptosporidium* infection was not analyzed.

## 5 Conclusion

In conclusion, the molecular characterization of *Cryptosporidium* spp. in Bactrian camels was studied across different regions, age groups, and seasons in Yili Prefecture of Xinjiang. The results revealed a highly significant difference in infection rates among different counties and seasons (*p* < 0.001), while the difference among different age groups was not significant (*p* > 0.05), indicating the presence of diverse epidemic characteristics of *Cryptosporidium* in camels in Yili Prefecture. However, due to the narrow age range in this study, further research with a broader age range is needed. Three species of *Cryptosporidium* that are detected in camels are zoonotic, posing a potential threat to humans and underscoring the need for increased attention. This study only focused on *Cryptosporidium* infection in camels, and future research should investigate the infection status of *Cryptosporidium* spp. in the surrounding environment of camels and farmers involved in camel husbandry, aiming to effectively control *Cryptosporidium* infection in camels under the One Health perspective.

## Data availability statement

Publicly available datasets were analyzed in this study. This data can be found here: https://www.ncbi.nlm.nih.gov/. The unique sequences identified in this study have been deposited in the GenBank database under accession numbers PP851037 to PP851049 (ssu rRNA gene) and PP858856 to PP858859 (gp60 gene).

## Ethics statement

The protocol for this study was approved by the Animal Care and Use of Chinese Academy of Agricultural Sciences, and authorized by the Animal Ethical Committee of Shanghai Veterinary Research Institute. All fecal samples obtained from camel farms were obtained with permission from the farm owners.

## Author contributions

RM: Funding acquisition, Writing – original draft. AS: Investigation, Resources, Writing – original draft. YW: Investigation, Resources, Writing – original draft. CX: Methodology, Supervision, Writing – original draft. WT: Investigation, Methodology, Writing – original draft. HG: Data curation, Software, Writing – original draft. YH: Data curation, Formal analysis, Writing – original draft. YZ: Formal analysis, Software, Writing – original draft. GY: Conceptualization, Writing – review & editing. ZC: Conceptualization, Funding acquisition, Project administration, Writing – review & editing.

## Footnotes

^1^ https://data.stats.gov.cn/

^2^ http://tjj.xinjiang.gov.cn

^3^ http://blast.ncbi.nlm.nih.gov/Blast.cgi

^4^ https://www.megasoftware.net

^5^ https://xjyl.gov.cn/xjylz/c112409/201603/7c7a9d04d7a540f7994bb68db81ead00.shtml
